# Regional Differences in AIDS and Non-AIDS Related Mortality in HIV-Positive Individuals across Europe and Argentina: The EuroSIDA Study

**DOI:** 10.1371/journal.pone.0041673

**Published:** 2012-07-23

**Authors:** Joanne Reekie, Justyna D. Kowalska, Igor Karpov, Jurgen Rockstroh, Anders Karlsson, Aza Rakhmanova, Andrzej Horban, Ole Kirk, Jens D. Lundgren, Amanda Mocroft, for EuroSIDA in EuroCoord

**Affiliations:** 1 Medical School, University College London, London, United Kingdom; 2 Copenhagen HIV Programme, University of Copenhagen, Copenhagen, Denmark; 3 Belarus State Medical University, Minsk, Belarus; 4 University of Bonn, Bonn, Germany; 5 Venhaelsan-Sodersjukhuset, Stockholm, Sweden; 6 Medical Academy Botkin Hospital, St Petersburg, Russia; 7 Warsaw Medical University, Hospital of Infectious Diseases, Warsaw, Poland; 8 Department of Infectious Diseases, Rigshospitalet, Copenhagen, Denmark; Rollins School of Public Health, Emory University, United States of America

## Abstract

**Background:**

Differences in access to care and treatment have been reported in Eastern Europe, a region with one of the fastest growing HIV epidemics, compared to the rest of Europe. This analysis aimed to establish whether there are regional differences in the mortality rate of HIV-positive individuals across Europe, and Argentina.

**Methods:**

13,310 individuals under follow-up were included in the analysis. Poisson regression investigated factors associated with the risk of death.

**Findings:**

During 82,212 person years of follow-up (PYFU) 1,147 individuals died (mortality rate 14.0 per 1,000 PYFU (95% confidence interval [CI] 13.1–14.8). Significant differences between regions were seen in the rate of all-cause, AIDS and non-AIDS related mortality (global p<0.0001 for all three endpoints). Compared to South Europe, after adjusting for baseline demographics, laboratory measurements and treatment, a higher rate of AIDS related mortality was observed in East Europe (IRR 2.90, 95%CI 1.97–4.28, p<.0001), and a higher rate of non-AIDS related mortality in North Europe (IRR 1.51, 95%CI 1.24–1.82, p<.0001). The differences observed in North Europe decreased over calendar-time, in 2009–2011, the higher rate of non-AIDS related mortality was no longer significantly different to South Europe (IRR 1.07, 95%CI 0.66–1.75, p = 0.77). However, in 2009–2011, there remained a higher rate of AIDS-related mortality (IRR 2.41, 95%CI 1.11–5.25, p = 0.02) in East Europe compared to South Europe in adjusted analysis.

**Interpretations:**

There are significant differences in the rate of all-cause mortality among HIV-positive individuals across different regions of Europe and Argentina. Individuals in Eastern Europe had an increased risk of mortality from AIDS related causes and individuals in North Europe had the highest rate of non-AIDS related mortality. These findings are important for understanding and reviewing HIV treatment strategies and policies across the European region.

## Introduction

Mortality in HIV-positive individuals continues to decrease due to improvements in combination antiretroviral therapy (cART) and its availability, although it remains higher than in the general population [1]. A high proportion of the mortality observed in HIV-positive individuals is now due to non-AIDS related causes [2], [3]. However, the incidence of non-AIDS related mortality, from causes such as cardiovascular disease (CVD), liver disease, and cancer, has also declined [3]. Drug specific toxicity, lifestyle factors (smoking drug and alcohol misuse), co-infection with other viruses (hepatitis B and C, human papillomavirus) and residual immunodeficiency are all thought to contribute to the higher relative risk of non-AIDS related mortality observed in HIV-positive individuals compared to the general population [4].

Research investigating regional differences across Europe, particularly allowing a comparison with East Europe, is limited. In Western and Central Europe, widespread access to antiretroviral therapy has led to considerable drops in AIDS related mortality [5]. However, in Eastern Europe, a region with one of the fastest growing HIV epidemics worldwide [6], the uptake of cART remains low and previous studies have reported substantial differences in access to care and treatment compared to the rest of Europe [7], [8]. Shortly after the introduction of cART, the EuroSIDA study reported that the all-cause mortality rate in Western Central Europe was significantly lower than in North and South Europe [9]. Since then, the study has grown substantially, particularly in Eastern Europe where few cohort studies are established. Additionally, to allow for comparisons outside of Europe the study includes data from Argentina, where the uptake of cART is high, with more than 95% of those in need of cART (CD4 count <350 cells/mm^3^) receiving it [10]. The study provides a unique opportunity to establish whether, in the current treatment era, regional differences in the mortality rate of HIV-positive individuals remain.

## Methods

### Ethics Statement

Informed consent and ethics committee approval were obtained in each participating centre according to national guidelines. Full study details are available at www.cphiv.dk.

### Inclusion Criteria

The EuroSIDA study is a prospective study of individuals with HIV-1 in more than 100 centres across Europe, Israel and Argentina. To be eligible for inclusion into EuroSIDA, individuals must be aged 16 or older and have a pre-booked outpatient appointment at the centre. Starting in May 1994, there have been eight different periods of enrolment in EuroSIDA (cohorts I–VIII). Currently, 16,597 individuals are enrolled in EuroSIDA. The study has expanded into Eastern Europe with half of the individuals originating from Eastern European countries in Cohort VIII. Data is collected and updated at 6-monthly intervals on standardised recruitment and follow-up forms. Full study details are available at www.cphiv.dk. The incidence of LTFU in EuroSIDA has previously been reported to be fairly consistent over time at <5% per 100 PYFU [11]. An extensive quality assurance program has been established that includes data checking at the coordinating office, as well as regular monitoring visits with source verification of all new major events, plus a random selection of individuals followed at the clinics.

### Statistical Methods

Individuals were included from baseline, defined as either 1^st^ January 2002 or enrolment into EuroSIDA, whichever occurred later, this was when EuroSIDA expanded its network in East Europe. Additionally, previous EuroSIDA studies have reported mortality rates in earlier time periods [3], [12]. Follow-up was until death, or 6 months after their last recorded visit (to allow for delays in reporting death), whichever occurred first. The incidence of mortality was calculated per 1,000 person years of follow-up (PYFU) and stratified by region. Individuals were divided into 6 regions according to country of residence as follows:

- South Europe: Greece, Israel, Italy, Portugal, Spain.
- West Central Europe: Austria, Belgium, France, Germany, Luxembourg, Switzerland.
- North Europe: Denmark, Finland, Ireland, the Netherlands, Norway, Sweden, the United Kingdom
- East Central Europe: Bulgaria, Croatia, the Czech Republic, Hungary, Poland, Romania, Serbia, Slovakia
- Eastern Europe: Belarus, Estonia, Latvia, Lithuania, the Russian Federation, Ukraine
- Argentina

In order to compare the regions of Europe and Argentina, South Europe was chosen as the reference group as this group contained the largest number of individuals.

For individuals who died, date and cause of death are reported by the site investigator and, since 2004, a Coding Causes of Death in HIV (CoDe) case report form is additionally completed for each fatal case and CoDe methods used to determine the underlying cause of death [13]. Deaths reported without a known cause were classified as either unknown AIDS or unknown non-AIDS mortality according to earlier developed methods [14].

Poisson regression analyses were used to investigate factors associated with the incidence of mortality. Variables investigated were age, gender, ethnic origin, smoking status, anaemia, hypertension, diabetes, hepatitis B and C status, HIV transmission group, CD4 count, any previous AIDS defining illnesses and year of follow-up. Age and year of follow-up were treated as continuous variables. CD4 count was categorised (<200, 200–349, 350–500, ≥500 cell/mm^3^, missing) to allow for patients with missing values to remain in the analysis. Further treatment for HIV was split into 4 categories, not on cART, on cART with a viral load <500 copies/ml, on cART with a viral load ≥500 copies/ml, or on cART with no viral load measurement available. cART was defined as receiving ≥3 antiretrovirals. A cut-off of 500 copies/ml was chosen as this was lower limit of detection of the viral load assay for some individuals. Both current (time-updated) and baseline values were investigated for variables that could change over time. Factors that were significant in univariate analysis (p<0.1) were included in multivariate analyses.

Individuals were classed as Hepatitis B positive if they had a positive HBV surface antigen test recorded and Hepatitis C positive if they had a positive HCV antibody test. Hypertension was defined as a diastolic blood pressure ≥90 or a systolic blood pressure ≥140 (mmHg) or receiving anti-hypertensive medication. If a diagnosis of insulin dependent diabetes was reported by the investigating centre or if an individual was receiving anti-diabetic medication they were classed as diabetic. Anaemia was defined as a haemoglobin level ≤12 or ≤14 (mg/dl) for females and males respectively [15]. Smoking status was defined as either never, current, previous or unknown.

All analysis was performed using SAS 9.1 (SAS institute, Cary North Carolina, USA).

## Results

13,310 HIV-positive individuals were included in the analysis. There were clear differences in demographics across the regions (table 1). In East Europe, individuals were more likely to be younger (median age 30), female (42%) and infected via intravenous drug use [IDU] (49%) or heterosexual sex (41%). In contrast the majority of the individuals in West Central (45%), and North Europe (59%) were infected through homosexual sex. Similar baseline CD4 counts were observed across the regions. In South, West Central, North, East Central Europe and Argentina around 75–80% were on cART at baseline compared to only 30% in East Europe. In these individuals on cART, 62% in East Europe had a viral load <500 copies/ml compared to 70–85% in the other regions.

**Table 1:** Baseline characteristics (baseline was defined as either 1^st^ January 2002 or enrolment into EuroSIDA which ever occurred later).

			South Europe				West centralEurope				North Europe			East Central Europe				East Europe				Argentina			
Total (N,% of total)			3246		24.4%		2920		21.9%		2866		21.5%	1700		12.8%		2082		15.6%		496		3.7%	
Gender (N.%)	Male		2391		73.7%		2246		76.9%		2332		81.4%	1221		71.8%		1171		57.7%		308		62.1%	
Race (N.%)	White		2971		91.5%		2102		72.0%		2426		84.6%	1674		98.5%		2074		102.3%		489		98.6%	
Exposure Group	Homosexual		1101		33.9%		1307		44.8%		1693		59.1%	627		36.9%		154		7.6%		126		25.4%	
(N.%)	Injection DrugUser		949		29.2%		406		13.9%		317		11.1%	449		26.4%		996		49.1%		62		12.5%	
	Heterosexual		988		30.4%		849		29.1%		684		23.9%	490		28.8%		830		40.9%		292		58.9%	
Prior AIDS (N.%)			853		26.3%		951		32.6%		915		31.9%	424		24.9%		402		19.8%		161		32.5%	
Hepatitis B (N.%)	Negative		2186		67.3%		2308		79.0%		2339		81.6%	1408		82.8%		1653		81.5%		357		72.0%	
	Positive		162		5.0%		202		6.9%		206		7.2%	81		4.8%		112		5.5%		26		5.2%	
	Unknown		898		27.7%		410		14.0%		321		11.2%	211		12.4%		317		15.6%		113		22.8%	
Hepatitis C (N.%)	Negative		1595		49.1%		1982		67.9%		1839		64.2%	995		58.5%		699		34.5%		295		59.5%	
	Positive		755		23.3%		460		15.8%		329		11.5%	480		28.2%		1014		50.0%		91		18.3%	
	Unknown		896		27.6%		478		16.4%		698		24.4%	225		13.2%		369		18.2%		110		22.2%	
Hypertension	No		1629		50.2%		1136		38.9%		862		30.1%	1045		61.5%		1295		63.9%		292		58.9%	
	Yes		488		15.0%		538		18.4%		505		17.6%	327		19.2%		119		5.9%		42		8.5%	
	Unknown		1129		34.8%		1246		42.7%		1499		52.3%	328		19.3%		668		32.%9		162		32.7%	
Diabetes	No		2904		89.5%		2012		68.9%		2614		91.2%	1609		94.6%		2061		101.6%		489		98.6%	
	Yes		180		5.5%		148		5.1%		93		3.2%	44		2.6%		5		0.2%		5		1.0%	
	Unknown		162		5.0%		760		26.0%		159		5.5%	47		2.8%		16		0.8%		2		0.4%	
Anaemia	No		2194		67.6%		1904		65.2%		1642		57.3%	776		45.6%		197		9.7%		187		37.7%	
	Yes		782		24.1%		747		25.6%		911		31.8%	295		17.4%		110		5.4%		205		41.3%	
	Unknown		270		8.3%		269		9.2%		313		10.9%	629		37.0%		1775		87.5%		104		21.0%	
Smoking	Never	706		21.7%		765		26.2%		576		20.1%		416	24.5%		387		19.1%		146		29.4%		
	Current	1495		46.1%		1179		40.4%		1156		40.3%		872	51.3%		1142		56.3%		169		34.1%		
	Previous	623		19.2%		669		22.9%		643		22.4%		306	18.0%		369		18.2%		146		29.4%		
	Unknown	422		13.0%		307		10.5%		491		17.1%		106	6.2%		184		9.1%		35		7.1%		
On cART (N.%)		2558		78.8%		2170		74.3%		2286		79.8%		1269	74.6%		603		29.7%		367		74.0%		
On cART with viral load<500copies/ml(N, % on cART)		1795		70.2%		1732		79.3%		1927		84.3%		978	77.0%		371		61.5%		253		68.9%		
Missing viral load on cART (N, % on cART)		16		0.6%		5		0.2%		11		0.5%		20	1.6%		40		6.6%		9		2.5%		
Age (median, IQR)			40		35–46		42		37–49		43		37–50	35		30–42		30		25–36		37		31–43	
CD4 count (median, IQR)		450		291–650		432		286–621		420		279–598		373	242–543		407		259–575		342		201–510		
Missing CD4 count (N, %)		20		0.6%		10		0.3%		15		0.5%		6	0.4%		96		4.6%		4		0.8%		
Baseline date (median, IQR)		1/02		1/02–12/03		1/02		1/02–12/05		1/02		1/02–12/03		1/04	1/02–12/08		2/06		4/04–6/08		2/06		11/03–6/06		

**Figure 1:**
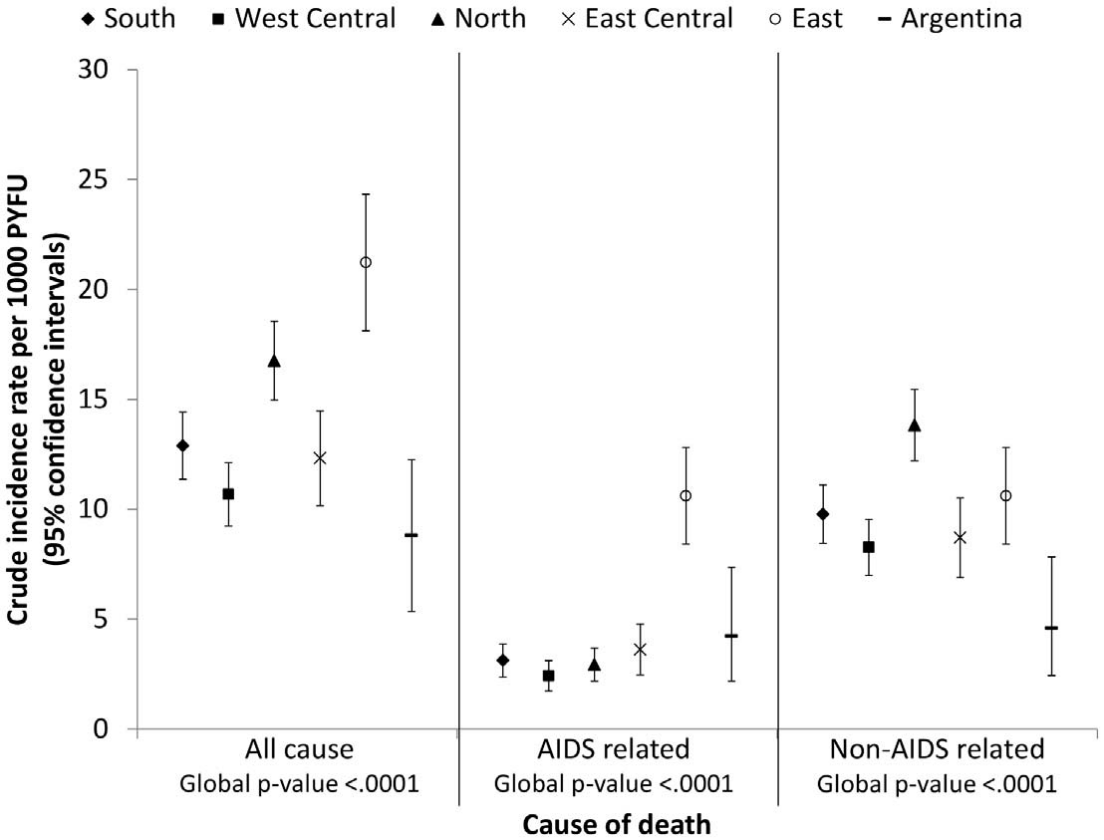
Crude mortality rate across the six different regions. Cause of death are reported by the site investigator and, since 2004, a Coding of Death in HIV (CoDe) case report form is additionally completed for each fatal case and CoDe methods used to determine the underlying cause of death.

From 2002 until a median last follow-up date of September 2011 (interquartile range [IQR] April 09-December 2011), individuals were followed for a total of 82,212 PYFU. During this time 1,147 patients died, the crude mortality rate was 14.0 per 1,000 PYFU (95% confidence interval [CI] 13.1–14.8). The majority (837, 73%) were attributed to non-AIDS related causes, the crude mortality rate was 10.2 non-AIDS related deaths per 1,000 PYFU (95% CI 9.5–10.9) compared to 3.8 AIDS related deaths per 1,000 PYFU (95% CI 3.4–4.2). Figure 1 shows the crude mortality rate for all cause, AIDS and non-AIDS related causes by region. Significant differences in mortality were seen across the 6 different regions (global p<0.0001 for all three endpoints). Higher all-cause mortality rates were observed in North and East Europe compared to the other regions. In East Europe a higher rate of AIDS related mortality was observed, the crude mortality rate due to AIDS related causes in East Europe was over three times higher than in South Europe (incidence rate ratio [IRR] 3.41, 95%CI 2.48–4.68, p<0001). In contrast, a higher rate of non-AIDS related mortality was observed in North Europe, compared to South Europe, the crude rate of non-AIDS related mortality in North Europe was 1.4 times higher (IRR 1.41, 95% CI 1.18–1.69, p<0001).

Table 2 shows the characteristics of the individuals who died at the time of their death in each of the 6 regions. Those in East Europe were younger at the time of their death and had lower CD4 counts. A higher proportion on individuals in East Europe (66%) and Argentina (68%) had experienced an AIDS event prior to their death than in the other regions. In contrast, in those dying in South, West Central, North, East Central Europe and Argentina around 30–40% had been diagnosed with a non-AIDS event prior to their death, whereas only 17% had been diagnosed in East Europe.

**Table 2:** Characteristics at time of death of 1147 patients who died between 2002–2011 across Europe and Argentina.

		Region						
		South	West Central	North	East Central	East	Argentina	P-value*
N died (% of total in each region)		273 (8.4%)	208 (7.1%)	337 (11.8%)	126 (7.4%)	178 (8.6%)	25 (5.0%)	<.0001
Age (median, IQR)		47 (42–57)	48 (43–59)	50 (43–59)	42 (36–54)	35 (30–41)	41 (34–48)	<.0001
CD4 count, cells/mm^3^ (median, IQR)		208 (78–392)	249 (85–450)	260 (113–452)	236 (90–416)	156 (45–296)	58 (24–136)	0.0002
Missing CD4 count (N, %)		56 (20.5%)	39 (18.8%)	78 (23.2%)	23 (18.3%)	82 (46.1%)	11 (44.0%)	
Ever started cART (N, %)		261 (95.6%)	195 (93.8%)	325 (96.4%)	112 (89.9%)	89 (50.0%)	24 (96.0%)	<.0001
HIV viral load <500 copies/ml (N,% with viral load measured)		128 (63.1%)	101 (61.6%)	187 (71.4%)	48 (58.5%)	17 (37.0%)	6 (60.0%)	0.0005
Missing viral load (N,%)		70 (25.6%)	44(21.2%)	75 (22.3%)	44 (34.9%)	132 (74.2%)	15 (60.0%)	
AIDS reported prior to death (N, %)		159 (58.2%)	123 (59.1%)	168 (49.9%)	75 (59.5%)	118 (66.3%)	17 (68.0%)	<.0001
Most recent AIDS diagnosis (N, % of AIDS diagnosis)								
Oesophageal candidiasis		12 (7.6%)	23 (18.7%)	32 (19.1%)	7 (9.3%)	26 (22.0%)	2 (11.8%)	
PCP		14 (8.8%)	9 (7.3%)	25 (14.9%)	7 (9.3%)	8 (6.8%)	2 (11.8%)	
HIV wasting syndrome		8 (5.0%)	12 (9.8%)	10 (6.0%)	6 (8.0%)	18 (15.3%)	1 (5.9%)	
Pulmonary TB		18 (11.3%)	5 (4.1%)	6 (3.6%)	7 (9.3%)	21 (17.8%	1 (5.9%)	
NHL		24 (15.1%)	10 (8.1%)	20 (11.9%)	8 (10.7%)	2 (1.7%)	1 (5.9%)	
Non-AIDS event prior to death (N,%)		109 (39.9%)	63 (30.3%)	107 (31.8%)	45 (35.7%)	31 (17.4%)	8 (32.0%)	<.0001
Non-AIDS events reported (N,%)								
NADM		49 (17.9%)	24 (11.5%)	45 (13.3%)	18 (14.3%)	4 (2.2%)	4 (16%)	
Cardiovascular disease		32 (11.7%)	30 (14.4%)	52 (15.4%)	19 (15.1%)	3 (1.7%)	1 (4%)	
Pancreatitis		10 (3.7%)	7 (3.4%)	7 (2.1%)	4 93.2%)	10 (5.6%)	0 (0%)	
Liver related event		25 (9.2%)	9 (4.3%)	11 (3.3%)	10 (7.9%)	17 (9.6%)	4 (16%)	
End-stage renal disease		10 (3.7%)	7 (3.4%)	5 (1.5%)	0 (0%)	1 (0.6%)	0(0%)	

* Chi-squared test for categorical variables and Kruskal-Wallis for continuous variables. IQR; Inter-quartile range, PCP: Pneumocystis jiroveci pneumonia, TB: mycobacterium tuberculosis, NHL: non-Hodgkin’s lymphoma, NADM: non-AIDS defining malignancy.

Table 3 gives the results of the Poisson regression analysis and presents the incidence rate ratios for all-cause, AIDS and non-AIDS related mortality for the five regions compared to South Europe. Adjusting for gender, age, HIV exposure group, AIDS diagnosis prior to baseline, non-AIDS event prior to baseline, hepatitis B and C status, hypertension, anaemia, baseline CD4 count, baseline date and baseline on cART/viral load status accounted for some of the higher rate of AIDS-related mortality observed in East Europe, though the rate of AIDS-related mortality was still more than double the rate in South Europe (IRR 2.27, 95%CI 1.42–3.46, p = 0.0006). Further, modelling age, hepatitis B and C status, hypertension, anaemia, CD4 count, year of follow-up and on cART viral load as time updated covariates the rate of AIDS related mortality was more than three times higher in East Europe compared to South.

**Table 3:** Results from univariate and multivariate Poisson regression analysis investigating mortality rates by region (South is the reference region).

All-cause mortality	Univariate						Multivariate^a^							Multivariate^b^					
Region	IRR			95% CI		p-value	IRR			95% CI			p-value	IRR		95% CI			p-value
South Europe	1.00			–		–	1.00			–			–	1.00		–			–
West Central Europe	0.83			0.69–0.99		0.04	0.99			0.82–1.20			0.90	0.85		0.70–1.03			0.09
North Europe	1.30			1.11–1.53		0.001	1.50			1.26–1.78			<.0001	1.37		1.15–1.62			0.0003
East Central Europe	0.96			0.77–1.18		0.67	1.31			1.05–1.64			0.01	1.28		1.03–1.59			0.02
East Europe	1.65			1.36–1.99		<.0001	2.07			1.57–2.73			<.0001	1.77		1.42–2.20			<.0001
Argentina	0.68			0.45–1.03		0.06	0.99			0.65–1.52			0.97	0.77		0.50–1.16			0.21
**AIDS related mortality**	**Univariate**						**Multivariate** c							**Multivariate** d					
**Region**	**IRR**		**95% CI**			**p-value**	**IRR**		**95% CI**			**p-value**		**IRR**	**95% CI**			**p-value**	
South Europe	1.00		–			–	1.00		–			–		1.00	–			–	
West Central Europe	0.77		0.53–1.13			0.18	0.84		0.57–1.23			0.36		0.72	0.49–1.06			0.09	
North Europe	0.94		0.66–1.34			0.73	1.09		0.75–1.58			0.66		1.01	0.70–1.46			0.95	
East Central Europe	1.16		0.78–1.74			0.46	1.29		0.84–1.97			0.24		1.43	0.93–2.19			0.10	
East Europe	3.41		2.48–4.68			<.0001	2.27		1.42–3.64			0.0006		2.90	1.97–4.28			<.0001	
Argentina	1.36		0.73–2.51			0.33	1.03		0.54–1.95			0.93		1.01	0.53–1.90			0.98	
**Non-AIDS related mortality**	**Univariate**						**Multivariate** a							**Multivariate** b					
**Region**	**IRR**	**95% CI**			**p-value**		**IRR**	**95% CI**			**p-value**			**IRR**	**95% CI**		**p-value**		
South Europe	1.00	–			–		1.00	–			–			1.00	–		–		
West Central Europe	0.85	0.69–1.04			0.11		1.01	0.81–1.26			0.92			0.88	0.70–1.10		0.27		
North Europe	1.41	1.18–1.69			0.0002		1.63	1.34–1.99			<.0001			1.51	1.24–1.82		<.0001		
East Central Europe	0.89	0.69–1.14			0.35		1.31	1.01–1.70			0.04			1.22	0.95–1.58		0.11		
East Europe	1.09	0.85–1.39			0.51		1.81	1.27–2.57			0.001			1.29	0.98–1.71		0.07		
Argentina	0.47	0.27–0.82			0.008		0.87	0.49–1.54			0.62			0.61	0.35–1.09		0.09		

a adjusted for baseline variables gender, age, HIV exposure group, hepatitis B and C status, prior AIDS diagnosis, prior non-AIDS diagnosis, hypertension, diabetes, anaemia, smoking status, CD4 count, baseline date and on cART viral load.

b adjusted as in a but age, hepatitis B and C status, hypertension, diabetes, anaemia, smoking status, CD4 count, year of follow-up and on cART viral load were included as time-updated covariates.

c adjusted for baseline variables gender, age, HIV exposure group, hepatitis B and C status, prior AIDS diagnosis, prior non-AIDS diagnosis, hypertension, diabetes, anaemia, CD4 count, baseline date and on cART viral load.

d adjusted as in a but age, hepatitis B and C status, hypertension, diabetes, anaemia, CD4 count, year of follow-up and on cART viral load were included as time-updated covariates.

A higher incidence of non-AIDS related mortality was observed, after adjusting for baseline variables (listed in the footnote of table 3), in North Europe (IRR 1.63, 95%CI 1.34–1.99, p<0001), East Central Europe (IRR 1.31, 95%CI 1.01–1.70, p = 0.04) and East Europe (IRR 1.81, 95%CI 1.27–2.57, p = 0.001) compared to South Europe (Table 3). After including time-updated covariates in the model the increased rate was no longer significant in East Central Europe (IRR 1.22, 95% CI 0.95–1.58, p = 0.11) or East Europe (IRR 1.29, 95% CI 0.98–1.71, p = 0.07) but there remained a significantly increased rate of mortality in North Europe (IRR 1.51, 95% CI 1.24–1.82, p<0001).

**Figure 2:**
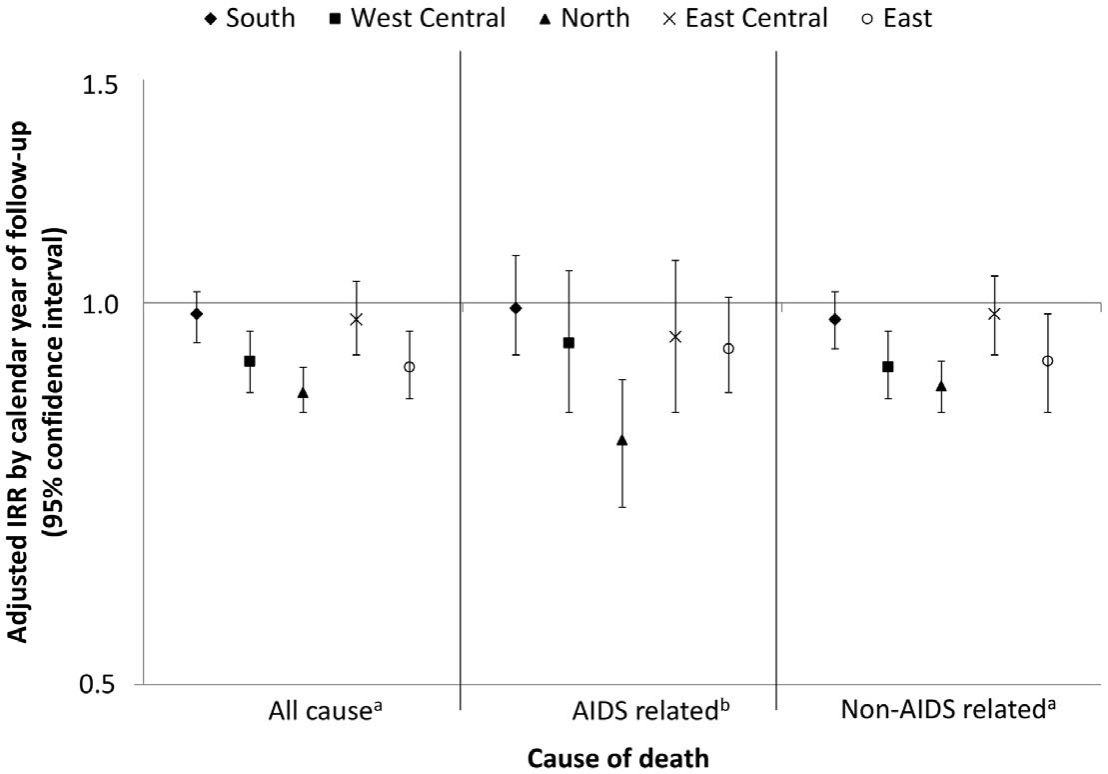
Incidence rate ratio (IRR) for all cause, AIDS and non-AIDS related mortality per calendar year of follow-up stratified by region. (Argentina was excluded from this analysis due to limited power). ^a^adjusted for gender, age*, HIV exposure group, hepatitis B* and C* status, prior AIDS diagnosis, prior non-AIDS diagnosis, hypertension*, diabetes*, anaemia*, smoking status*, CD4 count*, baseline date* and on cART viral load*. ^b^adjusted for gender, age*, HIV exposure group, hepatitis B* and C* status, prior AIDS diagnosis, prior non-AIDS diagnosis, hypertension*, diabetes*, anaemia*, CD4 count*, baseline date* and on cART viral load*. *time-updated.

In the adjusted analyses, increasing year of follow-up was associated with a significantly lower rate of both AIDS (IRR 0.91, 95%CI 0.87–0.95, p<.0001 and non-AIDS related mortality (IRR 0.90, 95%CI 0.88–0.92, p<0001). The test for interaction between region and calendar year of follow-up was significant for all-cause (p = 0.02) and AIDS related (p = 0.05) mortality, indicating that the effect of calendar year of follow-up on mortality rate may be different in each region, although it was non-significant for non-AIDS related mortality (p = 0.10). Figure 2 shows the adjusted incidence rate ratio for mortality per calendar year of follow-up stratified by region, from the fully adjusted model. Significant decreases over time in all-cause mortality rates were observed in West Central (IRR 0.90 per calendar year later, 95%CI 0.85–0.95, p = 0.0002), North (IRR 0.85 per calendar year later, 95%CI 0.82–0.89, p<0001), and East Europe (IRR 0.89 per calendar year later, 95%CI 0.84–0.95, p0.0005). No significant changes over time in all-cause mortality were observed in South (p = 0.32) or East Central Europe (p = 0.37). Argentina was excluded from this analysis due to the limited number of patients. Despite the observed decrease in mortality in East Europe over time, in the most recent period, 2009–2011, there remained a higher rate of all-cause (IRR 1.50, 95%CI 1.50–2.38, p = 0.08) and AIDS-related mortality (IRR 2.41, 95%CI 1.11–5.25, p = 0.02) compared to South Europe in adjusted analysis. However, in 2009–2011, the significantly higher rate in North Europe was no longer observed for all-cause (IRR 0.91, 95%CI 0.58–1.42, p = 0.18) or non-AIDS related mortality (IRR 1.07, 95%CI 0.66–1.75, p = 0.77), suggesting that the differences in North Europe, relative to South, are decreasing over time.

## Discussion

EuroSIDA is unique amongst studies of HIV-positive individuals in terms of its coverage across the entire continent of Europe. The results of this study with over 67,000 PYFU found significant differences in mortality rates across regions of Europe and Argentina. In particular, individuals in East Europe were found to have a higher mortality rate from AIDS related causes and individuals in North Europe a higher rate of non-AIDS related mortality. Similar rates of both AIDS and non-AIDS related mortality were seen across the other regions of Europe and Argentina. The mortality rate decreased over time particularly in West Central, North and East Europe.

Differences in the incidence of AIDS related mortality could partly be explained by variations in patient demographics including CD4 count and use of effective cART regimens. The epidemic in Eastern Europe is more recent, from 1995 there has been a rapid increase in HIV infections [16]. Since then a number of countries in the region have expanded access to antiretroviral coverage although treatment coverage remains low. UNAIDS estimated that in 2009 19% of those eligible for treatment in East Europe were accessing it, an estimated increase of 34% since the previous year [16]. In our study, after adjustment, the rate of AIDS related mortality appeared to be decreasing over time in East Europe, which may reflect improvements in antiretroviral coverage. However, after adjustment for time-updated CD4 count, use of cART, viral load and other factors, regional differences in AIDS related mortality were still significant, indicating that the differences cannot be fully explained by those variables collected and included in our models.

A high proportion of patients in East Europe are infected via IDU [17]. This was accounted for in the adjusted analysis but, IDU populations may differ across the regions. Several factors have been identified as contributing to the excess risk of mortality observed in IDUs in the cART era including decreased access and adherence to cART [18], [19], and more comorbidities such as co-infection with hepatitis C [20], [21]. In West Central Europe coverage of HIV services in IDU populations has been reported to be high, particularly access to cART [22]. However in East Europe services are low, there are low levels of needle exchange programmes [23] and very few IDUs have been found to be receiving antiretroviral treatment [22]. Opiate substitution programs are available in most countries but may be very limited in East Europe. Detailed information on opiate substitution programmes or whether individuals were actively injecting drug users was not available in this analysis. However, a sensitivity analysis, excluding those where the reported transmission route was IDU, found a similar increased incidence rate for East compared to South Europe, indicating that the higher prevalence of IDU does not account for the excess risk.

State-of-the-art care of HIV patients requires the utilisation of multiple health care interventions, such as the procurement of antiretroviral drugs, laboratory equipment, proper monitoring of antiretroviral treatment effectiveness and safety, and health care infrastructure in general [16]. Not all differences in patient care will have been captured by the use of effective cART, and overall lower levels of patient care in East Europe, may play a significant role in the higher rate of AIDS related mortality. Socio-economic and infrastructure aspects, for which we do not have data [24], may also play a role.

An increased rate of non-AIDS mortality was found in patients in North Europe compared to South Europe, and these differences could not be explained by adjusting for differences in patient demographics, treatment or other risk factors that were measured such as smoking status, co-infection and CVD risk factors, either at baseline or time-updated. However, the rate of non-AIDS related mortality does appears to be decreasing over time in North Europe with no difference observed in the most recent time period. The differences in non-AIDS mortality are probably not driven by poor patient care but by underlying differences in population morbidity and mortality. Smoking and excessive alcohol are found at an increased level in the HIV-positive population compared to the general population [25], [26] and could not be fully adjusted for in these analyses. Further, Mediterranean diet has been found to be associated with a lower risk of all-cause mortality, in particular due to CVD and cancer [27]. A recent study found that middle-aged men in North Europe had a 5% and woman 28% higher risk of all-cause mortality compared to South [28]. Although we have accounted for CVD factors such as diabetes and hypertension, these data were not available for all patients. Additionally, data on alcohol use was not available for this analysis although it has recently been added to the EuroSIDA follow-up forms. Furthermore, if HIV infection is accelerating the aging process [29], certain factors in North Europe such as a high intake of animal fat, smoking, and alcohol use, may be acting with this and could partly explain the differences observed, although we found no evidence that the relationship between age and mortality differed between regions (data not shown).

Another possibility is that the higher observed rate in North Europe is due to under ascertainment or incomplete reporting of mortality in other regions. Centres in some countries may be linked to national death registers allowing more complete reporting of mortality or have better methods of finding patients lost to follow-up (LTFU). LTFU in EuroSIDA is low, with individuals in North and West Central Europe having the lowest and those in East Europe the highest rates of LTFU in the study [11]. To investigate biases caused by under ascertainment of death we varied the right censoring interval from 6 months after last follow-up visit to 3 months and 1 year. The results remained consistent with the main analysis, suggesting that these differences aren’t solely due to underreporting in other regions. An analysis comparing the general HIV-negative population in these regions may also help clarify the reasons for these differences.

Demographically, individuals enrolled in the EuroSIDA study are fairly representative of each region based on comparisons with data from UNAIDS [30]. However, centres included in EuroSIDA are often university-associated clinics in larger cities, thus individuals in these clinics may have better access to care and may not be representative of all clinics in Europe, with potentially better clinical outcomes. This raises most concern in Eastern Europe, where the least favourable outcomes were seen, suggesting that improvements in this region are urgently needed. Further, within each of the regions some countries maybe quite heterogeneous in terms of their political and economic situation, however, the groupings provide a valuable regional overview of the differences in mortality rate across Europe.

In conclusion, differences were observed in the rate of all-cause mortality among HIV-positive individuals across different regions of Europe. Individuals in Eastern Europe had an increased risk of mortality from AIDS related causes in part due to differences in use of effective cART. Individuals in North Europe had the highest rate of non-AIDS related mortality which could not easily be explained but did appear to decrease over time. These findings are important for understanding and reviewing HIV treatment strategies and policies across the European region.
